# Supplementary material for: Efficient and stable inverted perovskite solar cells enabled by homogenized PCBM with enhanced electron transport
Source: Nat Commun. 2024 Oct 23;15:9154. doi: 10.1038/s41467-024-53283-5 (PMC11499991; doi:10.1038/s41467-024-53283-5)
Supplement: Supplementary file 1 — Supplementary information [file 41467_2024_53283_MOESM1_ESM.pdf]

Supplementary Information

Efficient and stable inverted perovskite solar cells enabled by homogenized PCBM with enhanced electron transport

Cheng Gong[1,†], Haiyun Li[1,†], Zhiyuan Xu[1,†], Yuheng Li[2,†], Huaxin Wang[1], Qixin Zhuang,[1] Awen Wang[2], Zhijun Li[1], Zhihao Guo[1], Cong Zhang[1], Baiqian Wang[1], Xiong Li[2,]* and Zhigang Zang[1,]*

[1]College of Photoelectric Engineering, Chongqing University, Chongqing 400044, China

[2]Wuhan National Laboratory for Optoelectronics, Huazhong University of Science and Technology, Wuhan 430074, Hubei, China

[†]These authors contributed equally: Cheng Gong, Haiyun Li, Zhiyuan Xu, Yuheng Li

*Correspondence to: xiongli@hust.edu.cn (X. L.); zangzg@cqu.edu.cn (Z. Z.)

Table of Contents

1. Supplementary Figures..3

2. Supplementary Tables...21

1. Supplementary Figures

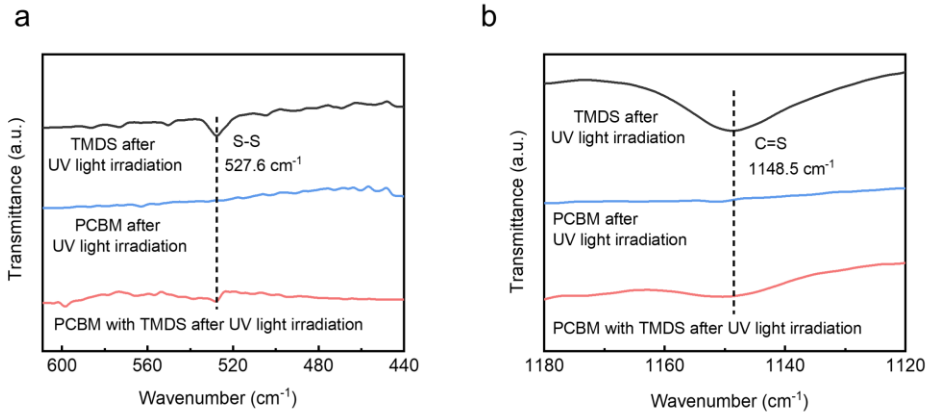

Supplementary Fig. 1. a, b Fourier transforms infrared (FTIR) spectra of TMDS and PCBM films without and with TMDS.

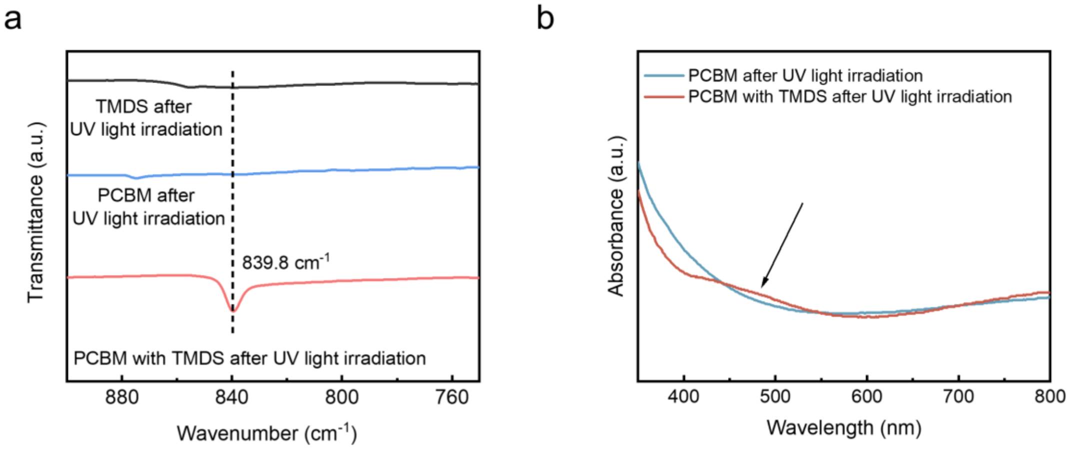

Supplementary Fig. 2. a, FTIR spectra of TMDS and PCBM films without and with TMDS. **b,** UV–vis absorption spectra of the PCBM and PCBM with TMDS film.

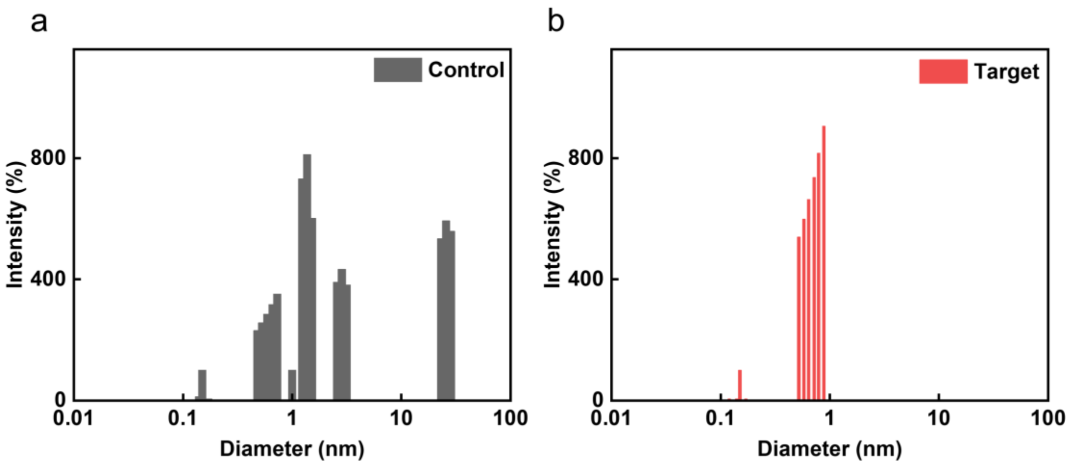

Supplementary Fig. 3. a, **b** DLS spectra of the (**a**) pristine PCBM in chlorobenzene solution and (**b**) TMDS modified PCBM solution after UV light irradiation.

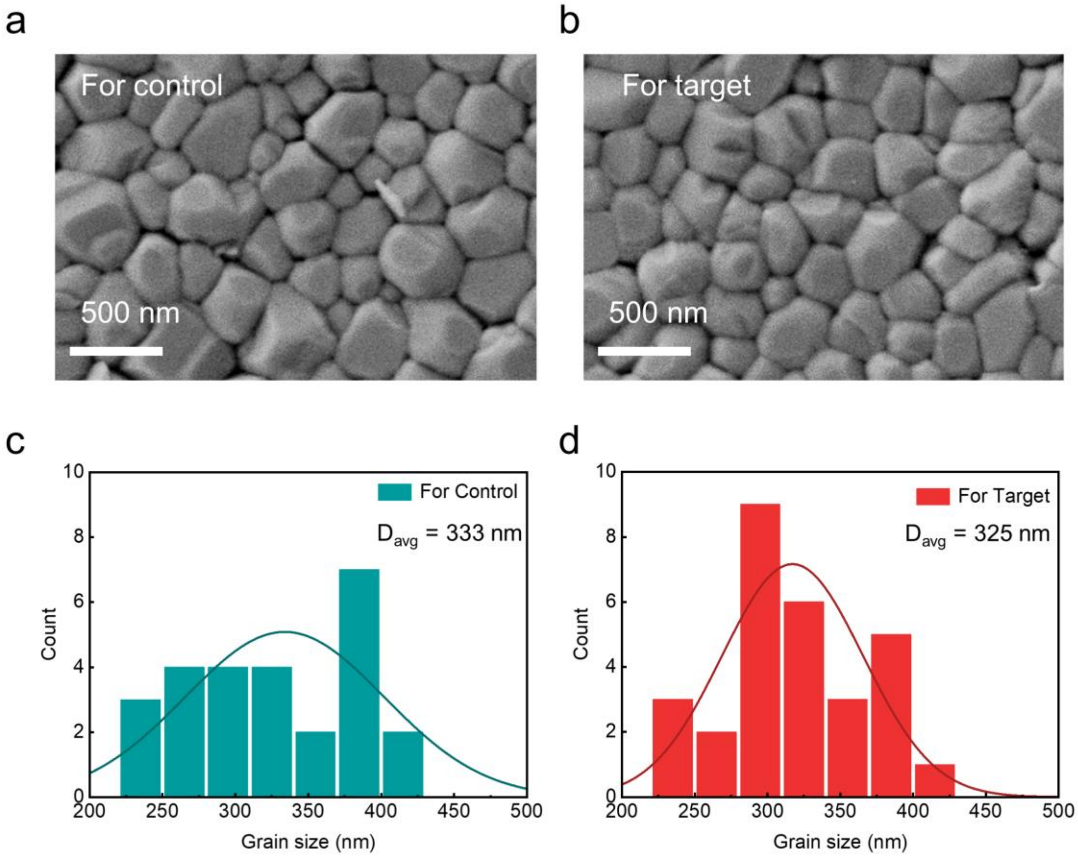

Supplementary Fig. 4. a, **b** The surface morphology and **c**, **d** grain size statistics of the perovskite films utilized for both control and target samples depicted in Fig. 2a.

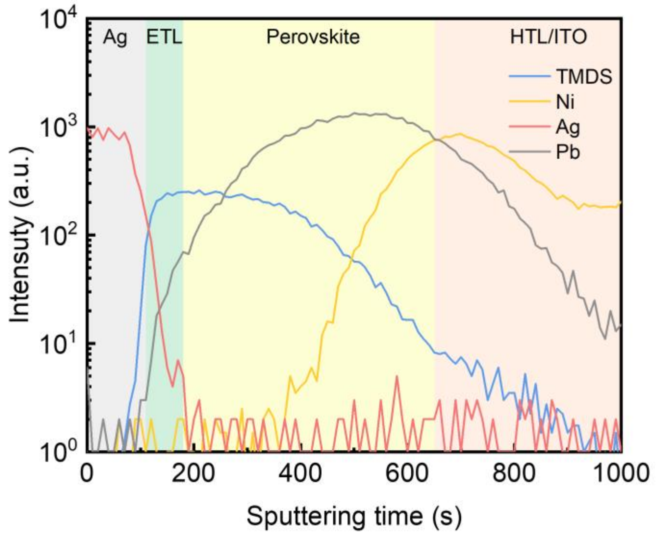

Supplementary Fig. 5. ToF-SIMS depth results for TMDS modified devices.

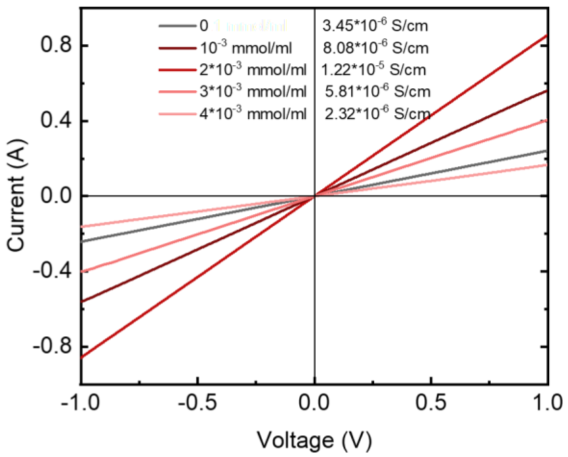

Supplementary Fig. 6. *I*–*V* curves of PCBM without and with different concentration of TMDS.

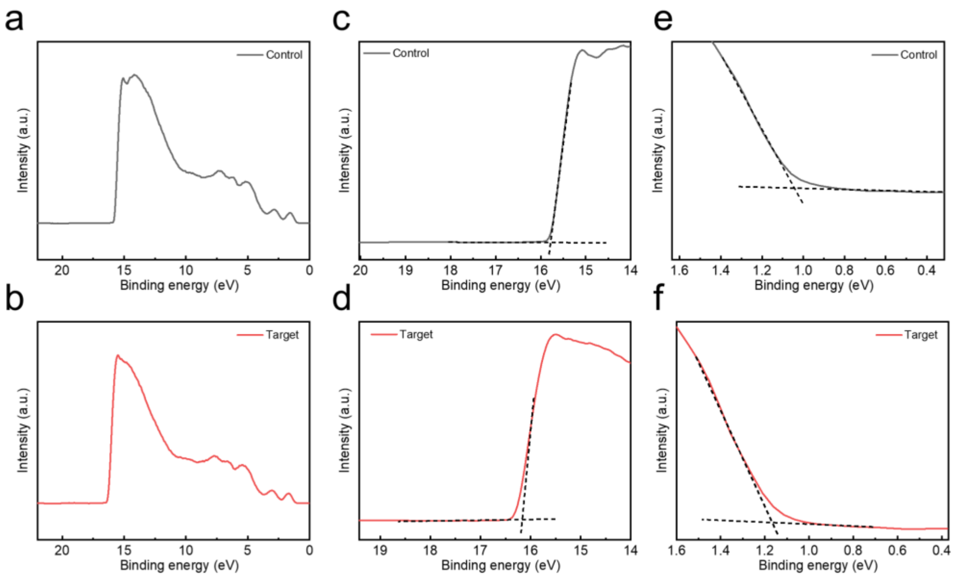

Supplementary Fig. 7. UPS measurements of the PCBM films **a**, **c, e** without and **b**, **d, f** with TMDS.

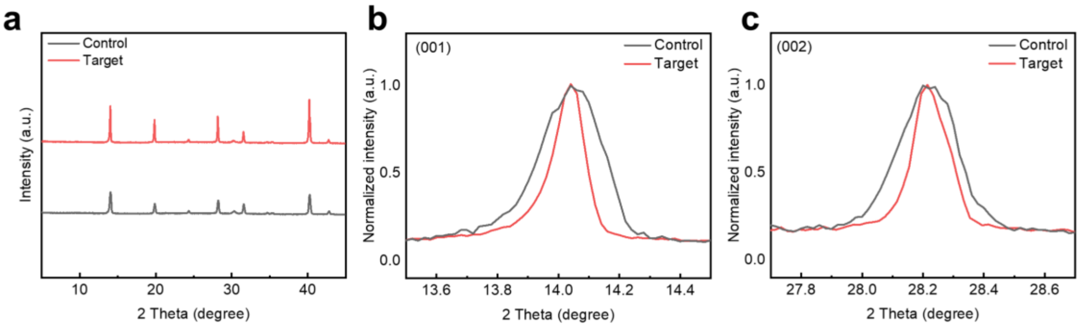

Supplementary Fig. 8. **a**, X-ray diffraction (XRD) patterns of the pristine and TMDS modified perovskite films. **b**, **c**, XRD patterns and full width at half maximum (FWHM) for control (**b**) and target (**c**) perovskite films.

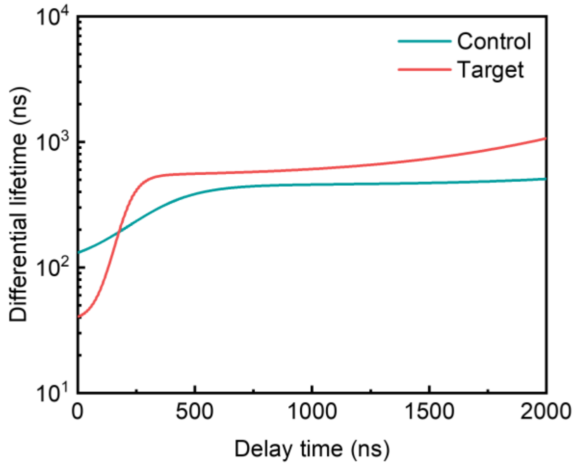

Supplementary Fig. 9. Calculated differential lifetimes from fits to the transients in Fig. 3i.

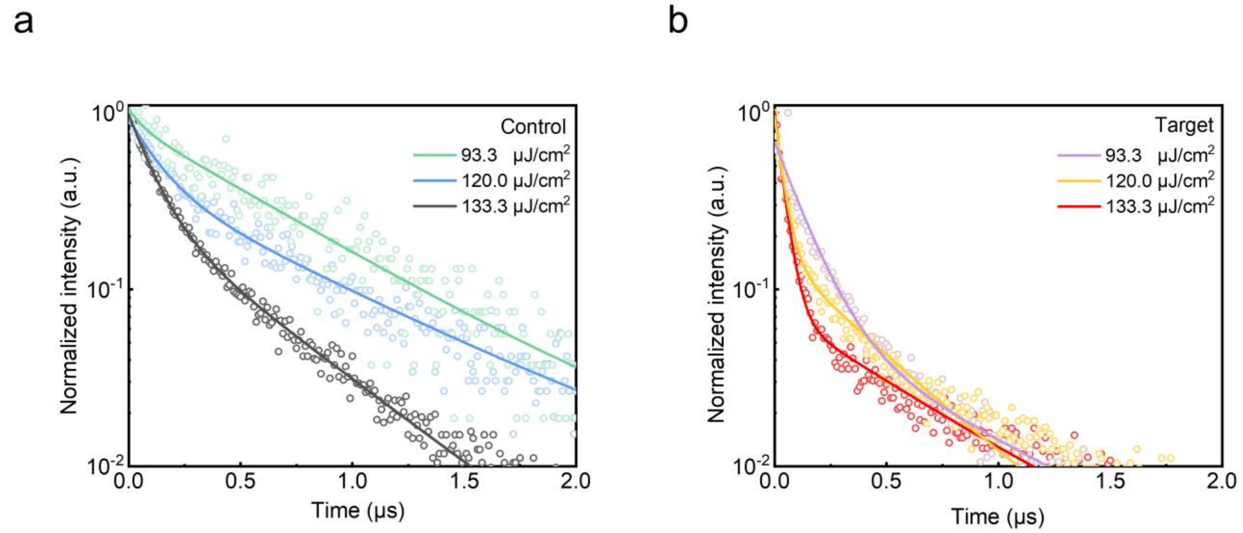

Supplementary Fig. 10. **a**, **b**, Intensity dependent time-resolved photoluminescence decays of the PVSK/PCBM (**a**) and PVSK/PCBM with TMDS (**b**).

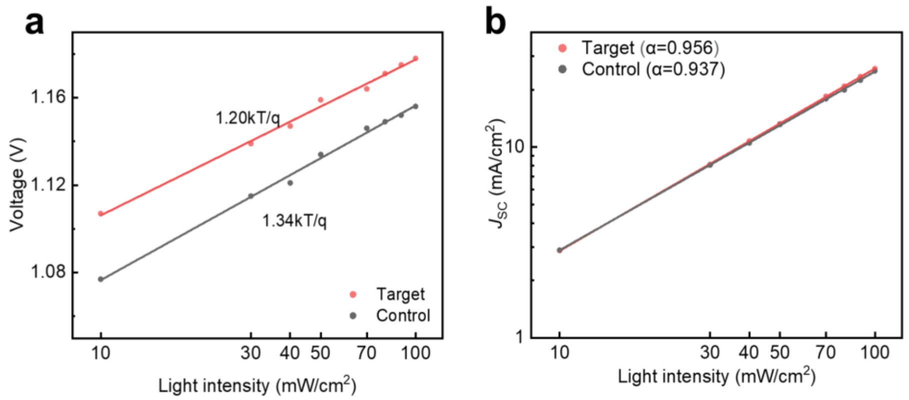

Supplementary Fig. 11. a, V_{OC} versus light intensity for the control and TMDS treated devices. **b**, The relationship between J_{SC} vs light intensity for the control and TMDS based devices.

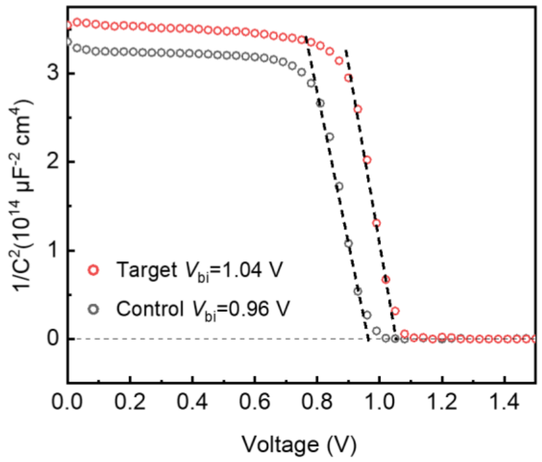

Supplementary Fig. 12. Mott–Schottky analysis for the PSCs without and with TMDS.

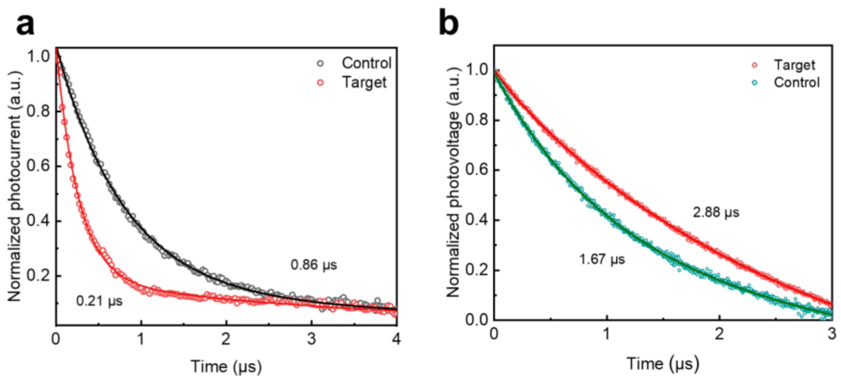

Supplementary Fig. 13. a, TPC for the control and modified PSCs. **b**, TPV for the control and modified PSCs.

a

中国认可
国际互认
检测
TESTING
CNAS L8490

230017163974

Test and Calibration Center of New Energy Device and Module, Shanghai Institute of Microsystem and Information Technology, Chinese Academy of Sciences (SIMIT)

Measurement Report

Report No. 23TR102511

**Client Name**	Huazhong University of Science and Technology
**Client Address**	1037 Luoyu Road, Wuhan, 430074, P. R. China
**Sample**	Perovskite Solar Cell
**Manufacturer**	Wuhan Photoelectric National Research Center
**Measurement Date**	25th October, 2023

**Performed by:**	Qiang Shi *Qiang Shi*	**Date:** 25/10/2023
**Reviewed by:**	Wenjie Zhao *Wenjie Zhao*	**Date:** 25/10/2023
**Approved by:**	Yucheng Liu *Yucheng Liu*	**Date:** 25/10/2023

Address: No.235 Chengbei Road, Jiading, Shanghai **Post Code:**201800

E-mail: solarcell@mail.sim.ac.cn **Tel:** +86-021-69976921

The measurement report without signature and seal are not valid.
This report shall not be reproduced, except in full, without the approval of SIMIT.

b

Report No. 23TR102511

Sample Information

Sample Type	Perovskite solar cell
Serial No.	10-1-1#
Lab Internal No.	23102501-11#
Measurement Item	I-V characteristic
Measurement Environment	23.9±2.0°C,41.3±5.0%R.H

Measurement of I-V characteristic

**Reference cell**	PVM 1121
**Reference cell Type**	mono-Si, WPVS, calibrated by NREL (Certificate No. ISO 2075)
**Calibration Value/Date of Calibration for Reference cell**	144.53mA/ Feb. 2023
**Measurement Conditions**	Standard Test Condition (STC):<br>Spectral Distribution: AM1.5 according to IEC 60904-3 Ed.3,<br>Irradiance: 1000±50W/m^2, Temperature: 25±2°C
**Measurement Equipment/ Date of Calibration**	AAA Steady State Solar Simulator (YSS-T155-2M) / July.2023<br>IV test system (ADCMT 6246) / June. 2023<br>Measuring Microscope (MF-B2017C) / July.2023<br>SR Measurement system (CEP-25ML-CAS) / April.2023
**Measurement Method**	I-V Measurement:<br>Logarithmic sweep in both directions (Voc to Isc and Isc to Voc) during one flash based on IEC 60904-1:2020.<br>Spectral Mismatch factor was calculated according to IEC 60904-7 and I-V correction according to IEC 60891.
**Measurement Uncertainty**	Area: 1.0%(k=2); Isc: 1.9%(k=2); Voc: 1.0%(k=2);<br>Pmax: 2.4%(k=2); Eff: 2.5%(k=2)

微系统 检测专

321

c

Report No. 23TR102511

====Measurement Results ====

	Forward Scan (Isc to Voc)	Reverse Scan (Voc to Isc)
Area	10.01 mm^2	
Isc	2.580 mA	2.580 mA
Voc	1.179 V	1.179 V
Pmax	2.559 mW	2.542 mW
Ipm	2.480 mA	2.487 mA
Vpm	1.032 V	1.022 V
FF	84.13 %	83.55 %
Eff	25.56 %	25.39 %

- Spectral Mismatch Factor: SMM=0.9950.
- Designated illumination area defined by a thin mask was measured by measuring microscope.
- Test results listed in this measurement report refer exclusively to the mentioned measured sample.
- The results apply only at the time of the test, and do not imply future performance.

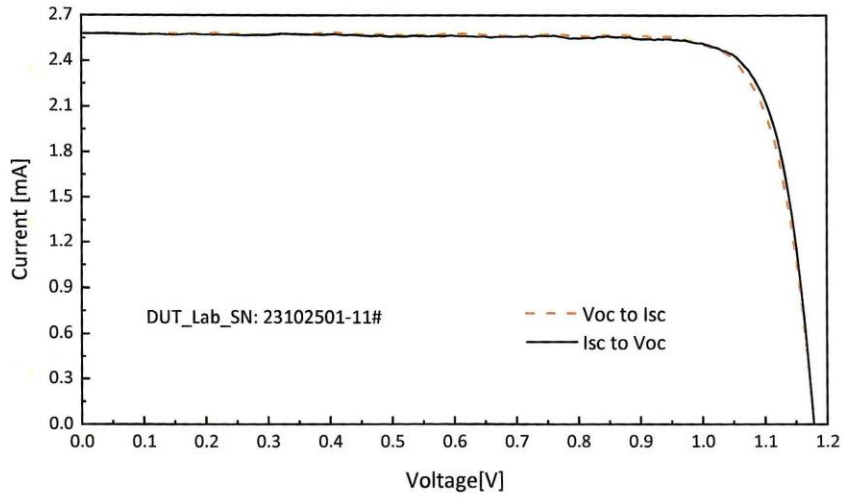

Fig.1 I-V curves of the measured sample

-------End of Report-------

3 / 3

Supplementary Fig. 14. a-c, Independent certification of one of the best-performing target devices by Shanghai Institute of Microsystem and Information Technology (SIMIT), Chinese Academy of Sciences.

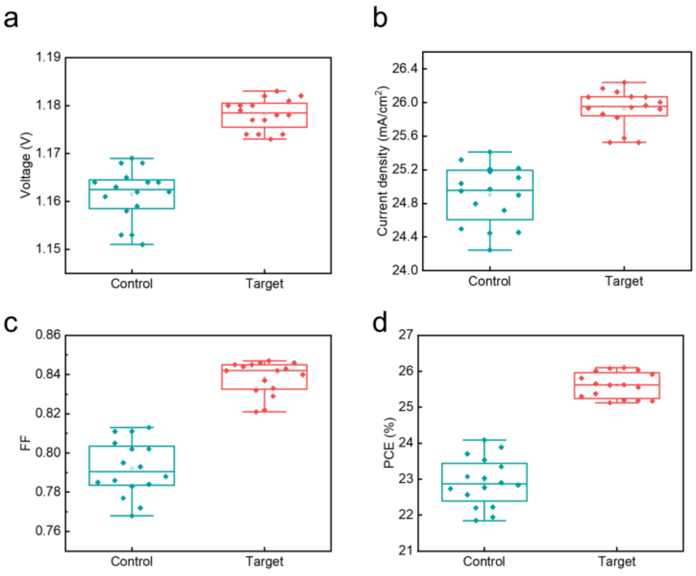

Supplementary Fig. 15. a-d, Statistical distribution diagram of the photovoltaic parameters of the control and target devices. The statistical data were obtained from 15 individual cells for each kind of device.

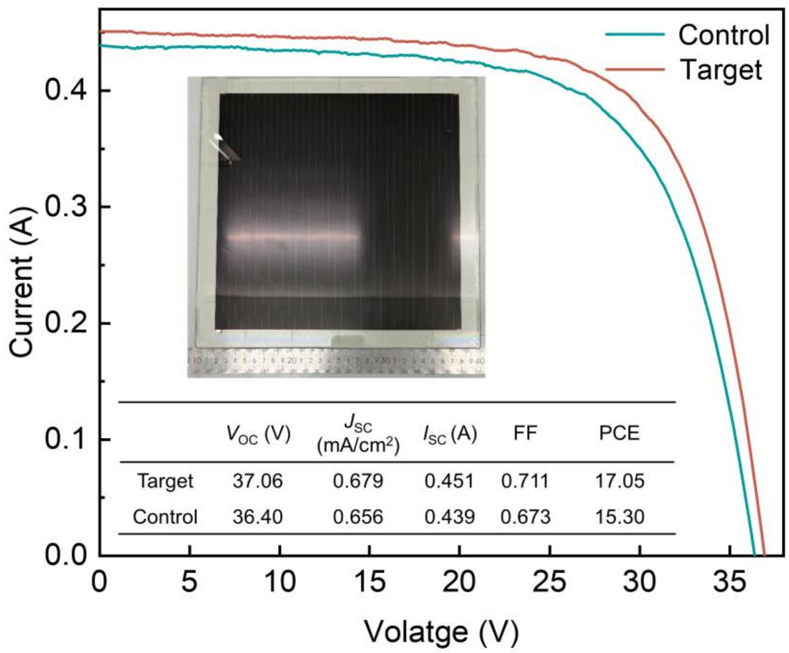

	V_{OC} (V)	J_{SC} (mA/cm^2)	I_{SC} (A)	FF	PCE
Target	37.06	0.679	0.451	0.711	17.05
Control	36.40	0.656	0.439	0.673	15.30

Supplementary Fig. 16. *J*–*V* curves of the module with relevant photovoltaic parameters and photographic image depicted in the insets.

2. Supplementary Tables

Supplementary Table 1. Calculated valence band (E_{VB}) and conduction band (E_{CB}) from $E_{cut\text{-}off}$, E_F and E_g for the PCBM films without and with TMDS.

Sample	$E_{cut\text{-}off}$ (eV)	$E_{on\text{-}set}$ (eV)	$E_{F\text{-}edge}$ (eV)	E_{VB} (eV)	E_g (eV)	E_{CB} (eV)
Target	16.17	1.17	-5.05	-6.22	2.16	-4.06
Control	15.79	1.04	-5.43	-6.47	2.16	-4.31

Supplementary Table 2. Fitting results of TRPL curves of the glass/PVSK without and with TMDS surface modified.

Sample	A_1	τ_1 (ns)	A_2	τ_2 (ns)	τ_{avg} (ns)
PVSK	0.77	147.9	0.23	750.6	511.0
PVSK/TMDS	0.69	239.5	0.31	1022.9	754.5

Note: The average weighted lifetime is extracted using the equation $\tau_{ave} = (A_1\tau_1^2 + A_2\tau_2^2)/(A_1\tau_1 + A_2\tau_2)$

Supplementary Table 3. Fitting results of TRPL curves of the glass/PVSK/PCBM without and with TMDS.

Sample	A_1	τ_1 (ns)	A_2	τ_2 (ns)	τ_{avg} (ns)
PVSK/PCBM	0.7	102.1	0.3	453.3	332.3
PVSK/PCBM with TMDS	0.93	37.5	0.07	527.0	289.1

Note: The average weighted lifetime is extracted using the equation $\tau_{ave} = (A_1\tau_1^2 + A_2\tau_2^2)/(A_1\tau_1 + A_2\tau_2)$

Supplementary Table 4. Summary of Photovoltaic parameters of the control and target devices.

Devices		J_{SC} (mA/cm^2)	V_{OC} (V)	FF	PCE (%)
Control	Average	24.90 ± 0.35	1.162 ± 0.006	0.792 ± 0.014	22.91 ± 0.67
	Champion	25.41	1.169	0.811	24.09
Target	Average	25.93 ± 0.22	1.178 ± 0.003	0.838 ± 0.009	25.60 ± 0.35
	Champion	26.07	1.182	0.847	26.10
